# *Staphylococcus aureus* Alpha Toxin Suppresses Effective Innate and Adaptive Immune Responses in a Murine Dermonecrosis Model

**DOI:** 10.1371/journal.pone.0075103

**Published:** 2013-10-02

**Authors:** Christine Tkaczyk, Melissa M. Hamilton, Vivekananda Datta, Xiang Ping Yang, Jamese J. Hilliard, Geoffrey L. Stephens, Agnieszka Sadowska, Lei Hua, Terrence O’Day, JoAnn Suzich, Charles Kendall Stover, Bret R. Sellman

**Affiliations:** 1 Department of Infectious Disease, MedImmune, LLC, Gaithersburg, Maryland, United States of America; 2 Pathology Department, MedImmune, LLC, Gaithersburg, Maryland, United States of America; 3 Department of Respiratory, Inflammatory and Autoimmune Diseases, MedImmune, LLC, Gaithersburg, Maryland, United States of America; 4 Biostatistics Department, MedImmune, LLC, Gaithersburg, Maryland, United States of America; Duke University Medical Center, United States of America

## Abstract

An optimal host response against *Staphylococcus aureus* skin and soft tissue infections (SSTI) is dependent on IL-1β and IL-17 mediated abscess formation. Alpha toxin (AT), an essential virulence factor for SSTI, has been reported to damage tissue integrity; however its effect on the immune response has not been investigated. Here, we demonstrate that infection with USA300 AT isogenic mutant (*Δhla*), or passive immunization with an AT neutralizing mAb, 2A3, 24 h prior to infection with wild type USA300 (WT), resulted in dermonecrotic lesion size reduction, and robust neutrophil infiltration. Infiltration correlates with increase in proinflammatory cytokines and chemokines, as well as enhanced bacterial clearance relative to immunization with a negative control mAb. In addition, infection with *Δhla,* or with WT +2A3, resulted in an early influx of innate IL-17^+^γδT cells and a more rapid induction of an adaptive immune response as measured by Th1 and Th17 cell recruitment at the site of infection. These results are the first direct evidence of a role for AT in subverting the innate and adaptive immune responses during a *S. aureus* SSTI. Further, these effects of AT can be overcome with a high affinity anti-AT mAb resulting in a reduction in disease severity.

## Introduction

*Staphylococcus aureus* is a leading cause of morbidity and mortality worldwide. While the majority of *S. aureus* infections are mild wound or skin and soft tissue infections, this pathogen can also cause invasive and life threatening infections such as bacteremia, sepsis, pneumonia, osteomyelitis and endocarditis [1]. These infections can be difficult to treat, due in part to an increasing incidence of antibiotic resistance [2]. As a result, new strategies of passive and active immunization targeting *S. aureus* virulence determinants are being explored to help combat these infections. Effective immunization strategies require a better understanding of how specific virulence factors facilitate *S. aureus* escape from the host immune response and potentiate disease [1], [3].

*S. aureus* alpha toxin (AT) is a cytolytic pore-forming toxin that has been demonstrated to play a key role in mouse and rabbit models of *S. aureus* disease (e.g. dermonecrosis, pneumonia, sepsis) [4]–[7]. Upon secretion, AT binds A-disintegrin and metalloprotease 10 (ADAM10) and forms heptameric pores in cell membranes leading to cell lysis and tissue damage [8]–[10]. In addition, AT activates ADAM10 mediated proteolysis of E-cadherin present in cell-cell adhesive contacts, leading to a disruption in epithelial and endothelial integrity which contributes to tissue damage and possibly bacterial dissemination [11], [12]. Mice deficient for ADAM10 expression in the skin are resistant to *S. aureus* infection providing evidence for the importance of AT and ADAM10 in the pathogenesis of *S. aureus* skin infection [13]. AT-deficient *S. aureus* mutants are also less virulent in animal infection models and methods of passive and active immunization targeting AT decrease skin lesion severity in *S. aureus* SSTI [5], [14]. These studies all demonstrate a major role for AT in *S. aureus* skin infection. However, it is unclear what impact AT has on the host immune response during a *S. aureus* SSTI.

Neutrophil infiltration and abscess formation are hallmarks of the host defense against *S. aureus* skin infections [15], [16]. In addition, γδ and CD4^+^ T cells have been reported to be important contributors to the immune response against a *S. aureus* cutaneous infection [16]–[18]. Recent publications have also described a critical role for IL-1β and IL-17 -mediated inflammatory responses ultimately leading to the expression of immune mediators including keratinocyte chemoattractant (KC), macrophage inflammatory protein-2 (MIP-2) and granulocyte monocyte colony stimulating factor (GM-CSF) required to attract circulating neutrophils into the site of the infection along with c-kit+-progenitor cells which differentiate into mature neutrophils in the tissue [19]–[23]. Upon arrival at the infection site, the activated neutrophils produce more cytokines including IL-1β which serve to mobilize additional neutrophils from the bone marrow. The resulting abscess then acts to limit the infection and ultimately clear the bacteria from the tissue.

Using an AT-deficient *S. aureus* USA300 strain (Δ*hla*) and an AT- neutralizing mAb, we investigated the role of AT in the pathogenesis of a *S. aureus* SSTI and its impact on the host immune response. Our results indicate that AT is necessary for *S. aureus* to effectively evade a protective immune response and that AT-mediated immune evasion can be inhibited with a specific mAb, thereby allowing the host innate and adaptive immune responses to respond appropriately and resolve the infection.

## Results

### Alpha Toxin Promotes Severe Skin Lesions and a Defect in Bacterial Clearance

**Figure 1:**
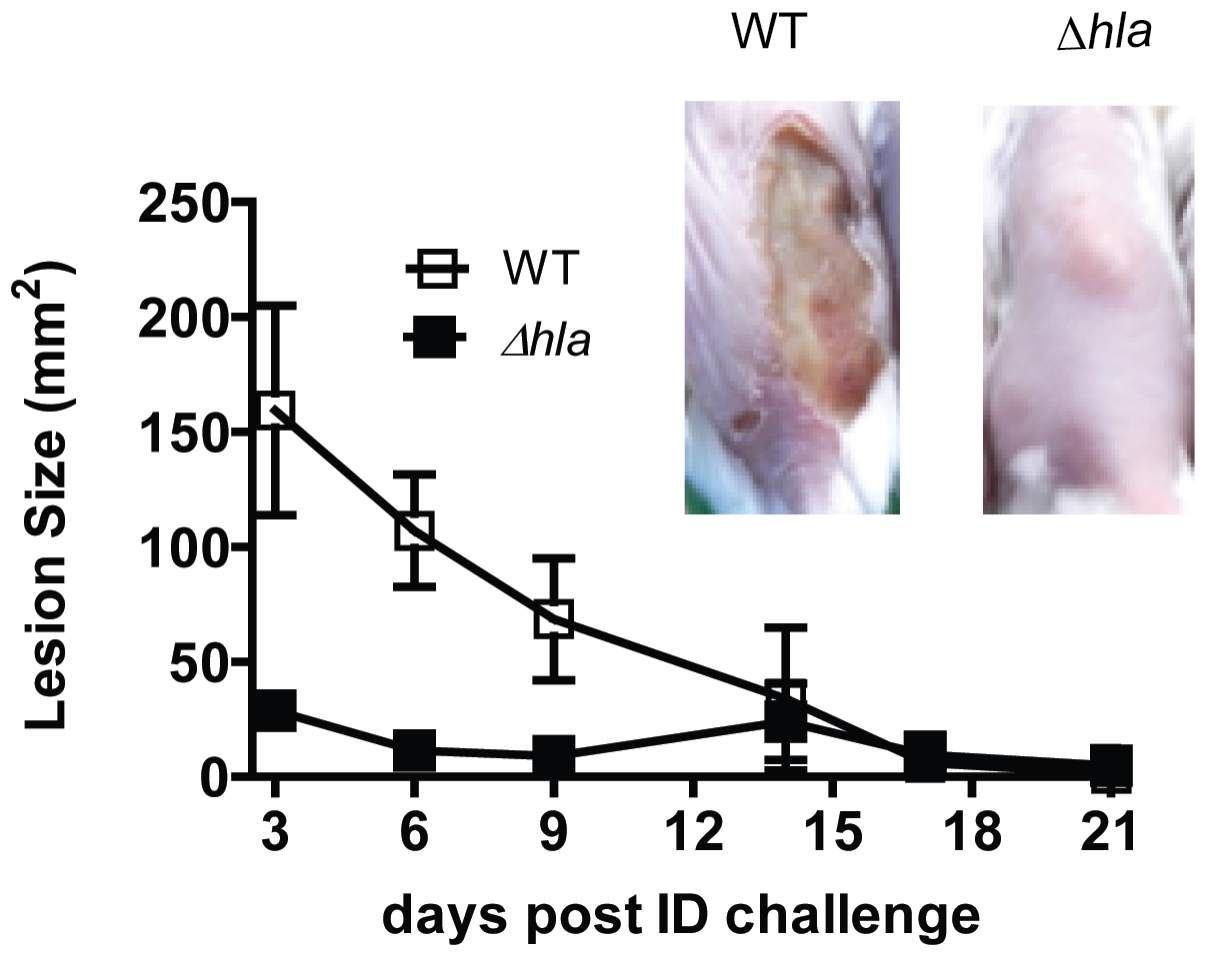
AT expression is necessary for *S. aureus* dermonecrosis. BALB/c mice (n = 10) were infected intradermally (ID) with 5×10^7^ cfu WT (□) or Δ*hla* (▪). Lesion sizes were measured up to 21 days post infection, and graphed as mean values ± standard deviation. Lesion sizes were significantly smaller for Δ*hla* up to day 10 (p≤ 0.007). Representative skin lesion pictures were taken on day 7.

**Figure 2:**
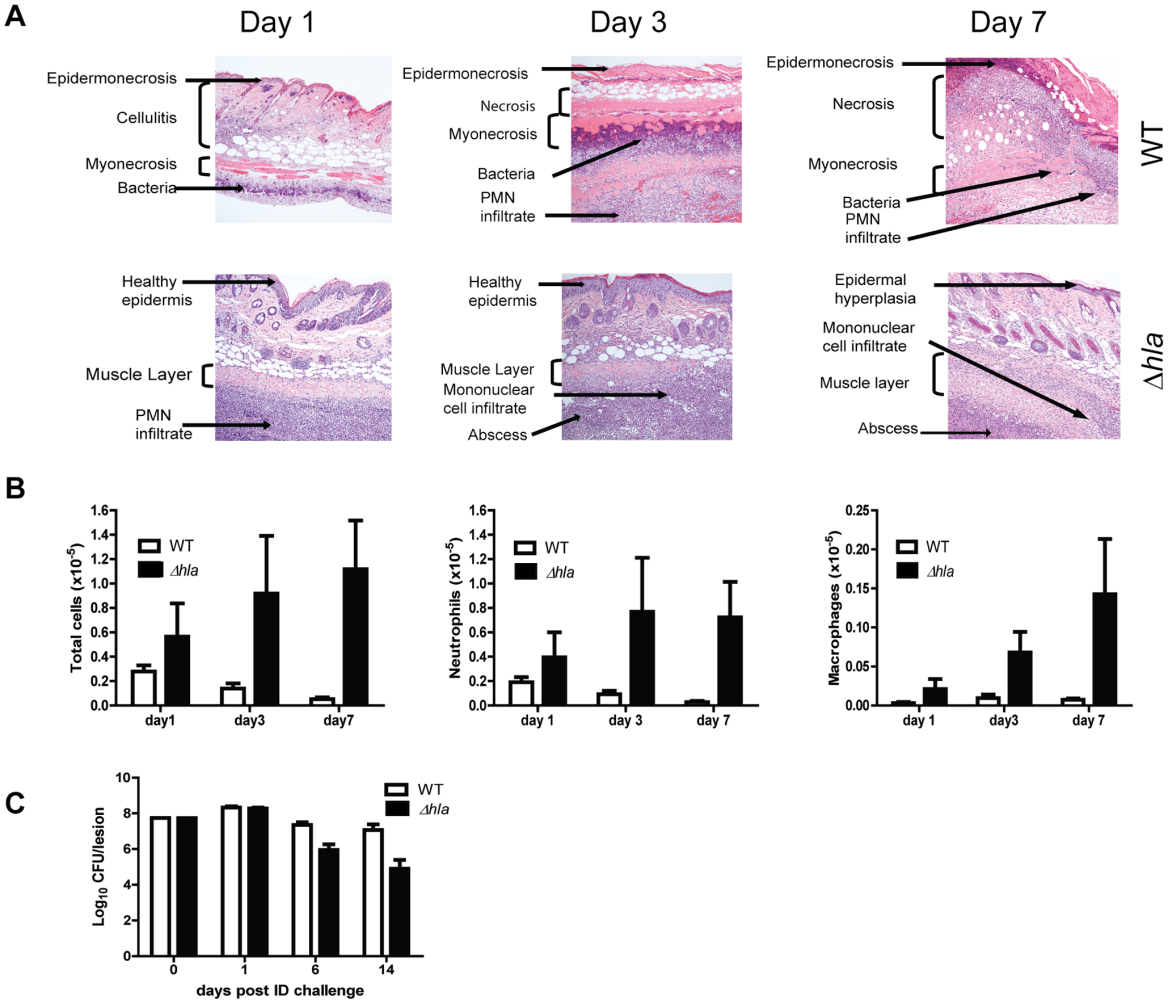
AT is a major contributor to disease severity in *S. aureus* dermonecrosis. BALB/c mice (n = 10) were infected ID with 5×10^7^ cfu WT (□) or Δ*hla* (▪). (A) Representative H&E stained histological sections 1, 3 and 7 days post-infection. (B) BALB/c mice (n = 5 for days1 and3, n = 10 for day 7) were infected ID with WT or Δ*hla*. Skin lesions were collected 1, 3 or 7 days post-infection and the neutrophils and macrophages were enumerated by flow cytometry as described in the methods. Cell number differences between WT and Δ*hla* infected mice were calculated with a Student’s t-test, and considered statistically difference if p<0.05 (indicated as*). (C) Bacterial CFU in skin lesions. Skins were harvested, homogenized for bacterial enumeration 1, 6, 9 and 14 days post infection (5 mice for days 1 and 6, 10 mice for days 9 and 14). Data were analyzed using a Mann-Whitney U test, and were statically significant after day 6 (p≤0.002).

To gain further insight into the mechanism by which AT potentiates *S. aureus* skin and soft tissue infections (SSTI), BALB/c mice were infected intradermally (ID) with *S. aureus* SF8300 wild type (WT) or its isogenic mutant SF8300 (Δ*hla*) deficient for AT expression. The resulting skin lesions were photographed, measured and subjected to histopathologic analysis. Animals infected with WT developed skin blisters within 8 hours post infection (data not shown) that became ulcerated lesions reaching a maximum size of 200 mm^2^ 48 h post infection. Over the 21 day experiment, lesion size decreased and they appeared to heal (Fig. 1). In contrast, 48 h post infection Δ*hla*-infected mice formed a raised lesion at the site of infection with little or no necrosis apparent on the skin surface measuring <10 mm^2^ (Fig. 1). Equally striking differences were apparent upon histological examination of the skin lesions (Fig. 2A). Twenty-four h post infection the mice infected with WT exhibited extensive necrosis of the skin and subcutaneous fatty tissue, severe muscle necrosis, and little or no inflammatory infiltrate. By day 7 the necrotic lesion became a large ulcer associated with extensive tissue necrosis and minimal submuscularis inflammatory infiltrate, granulation tissue formation and visible bacteria. In contrast, 24 h post infection Δ*hla*-infected mice exhibited little or no tissue damage along with extensive neutrophilic infiltration at the site of infection or abscess. The infection was contained within the granulation tissue formation. Additionally, there was morphological evidence of secondary healing, epidermal hyperplasia and wound repair on days 3 and 7. Consistent with a robust immune response, there were significantly greater numbers of neutrophils and macrophages in the mice infected with Δ*hla* relative to those infected with WT (Fig. 2B). These results suggest that AT not only plays a direct role in the tissue damage but also prevents the immune system from responding appropriately to a severe *S. aureus* SSTI. Consistent with this interpretation there was a significant reduction in bacterial numbers present in the lesions from animals infected with Δ*hla* relative to WT (Fig. 2C).

### Alpha Toxin Suppresses the Pro-inflammatory Cytokine and Chemokine Response

**Figure 3:**
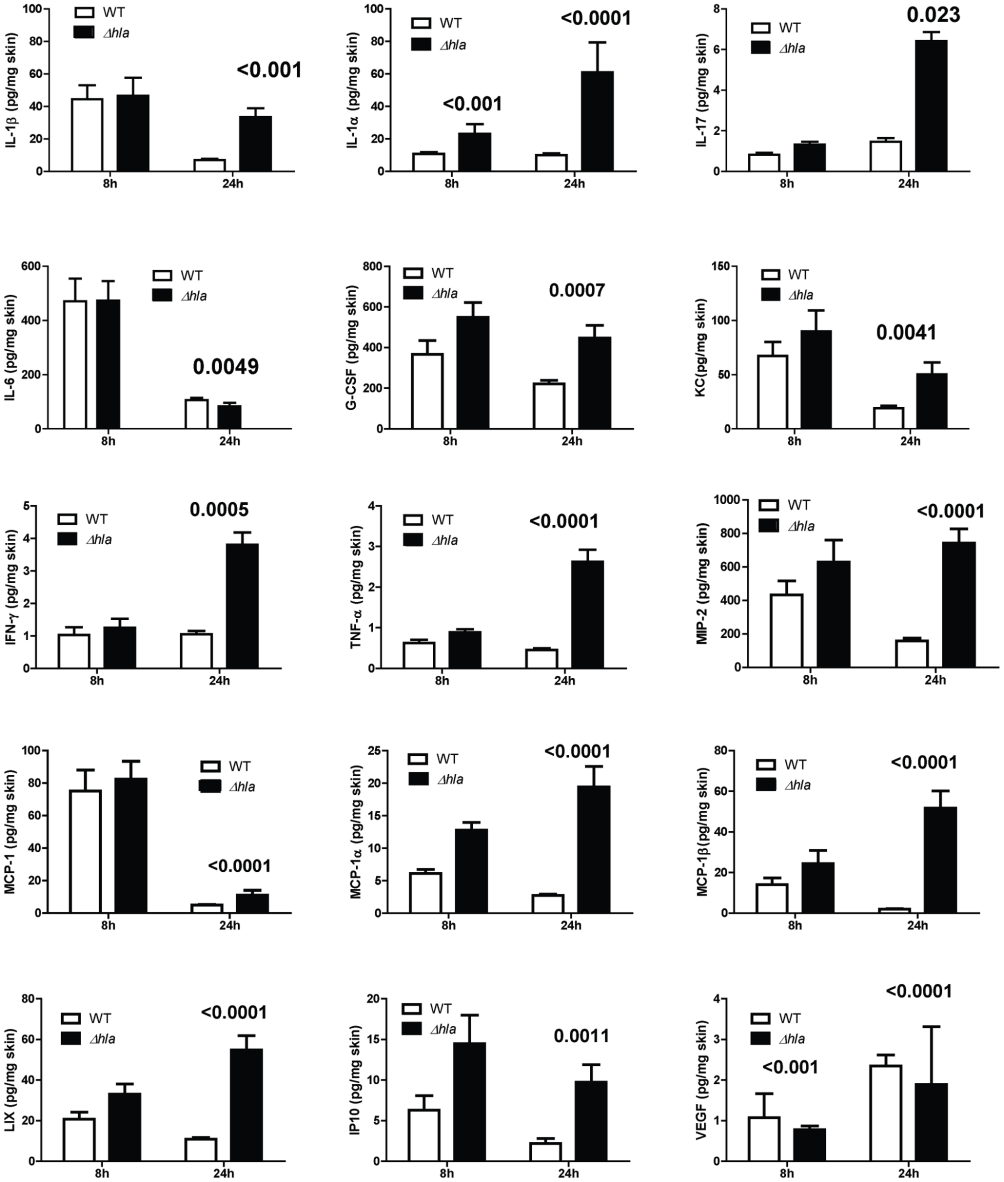
AT suppresses pro-inflammatory mediator production at infection site. BALB/c mice (n = 10) were ID infected with WT or Δ*hla* (5×10^7^ cfu in 50 µl PBS). Skin lesions were harvested after 8 or 24 h, homogenized and the homogenates analyzed by Luminex. Skin from uninfected mice (n = 5) was used as a control. Cytokine/chemokine levels represented as mean values ± standard deviation. Data were normalized in ng/mg of skin, and analyzed by a Student’s t-test. Values were considered statistically different if p≤0.05, and indicated on each graph.

The above results demonstrated reduced immune cell infiltration in mice infected with WT *S. aureus* expressing AT relative to mice infected with Δ*hla*. It has been reported that mice deficient in IL-1β or IL-17 are more susceptible to *S. aureus* skin infections due to a defect in neutrophil recruitment [20]–[23]. To determine if the increased PMN recruitment seen in mice infected with Δ*hla* was the result of a more robust proinflammatory mediator response, cytokine and chemokine levels were quantified from skin lesion homogenates of WT or Δ*hla*-challenged mice 8 and 24 h post infection. There were significantly higher levels of IL-1β, IFN-γ and IL-17, along with chemokines KC, MCP-1α/β, MIP-2, LIX, IP10 and G-CSF in the Δ*hla* infected skin lesions relative to WT (Fig. 3). Th2 cytokines IL-4 and IL-5 levels were below the limit of detection (data not shown). These results indicate that during a *S. aureus* skin infection AT impairs the host’s ability to initiate the proinflammatory cytokine and chemokine response necessary for infection resolution.

### Exogenous AT Increases Lesion Size and Reduces Immune Response to Δ*hla*

**Figure 4:**
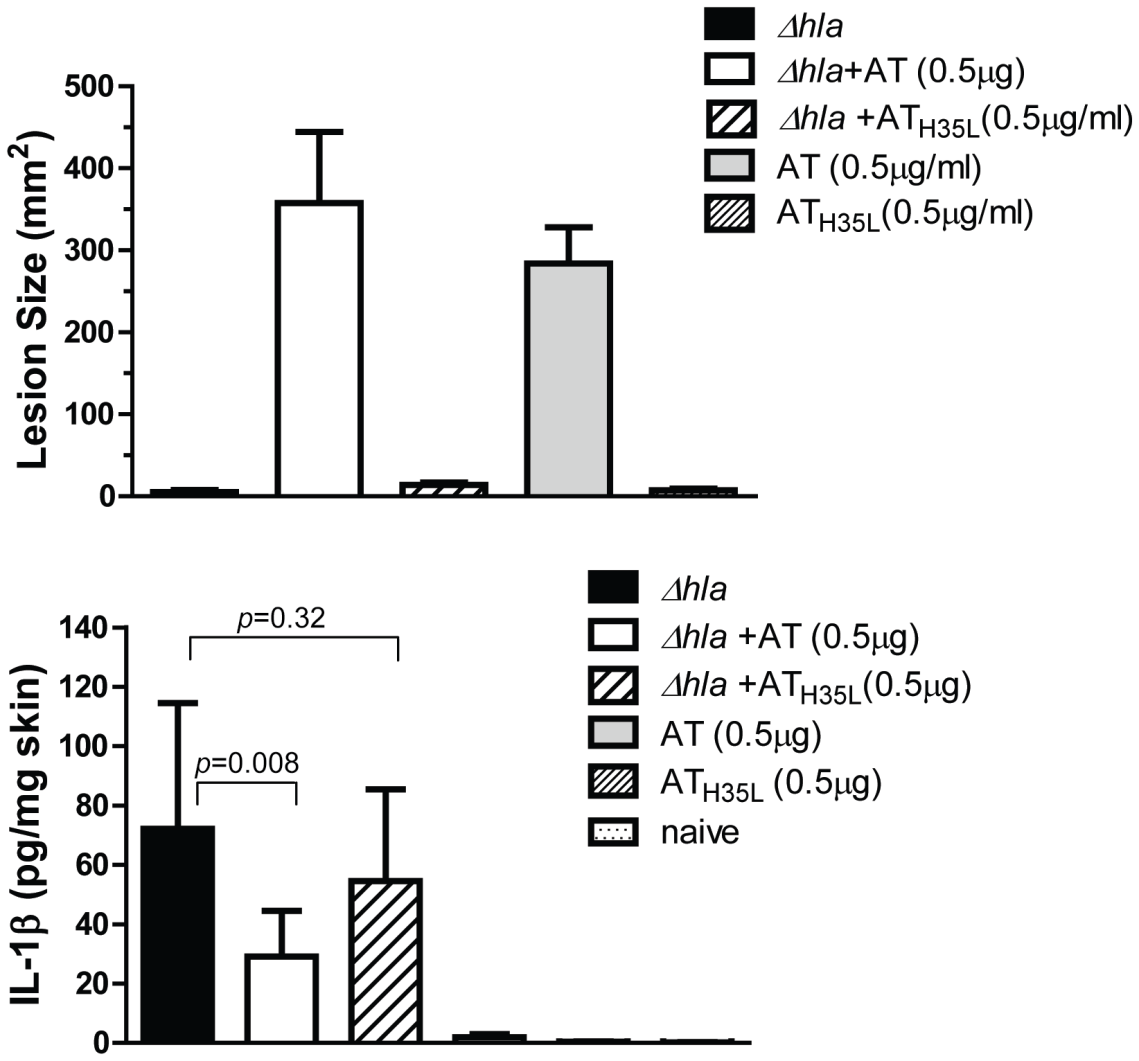
Sub-lytic AT dose potentiates Δ*hla*-mediated IL-1β production in skin lesions. BALB/c mice (n = 10) were ID challenged with Δ*hla* (5×10^7^ cfu), in combination with 0.5 µg AT or AT_H35L_. As controls, animals received each stimulus alone. (A) Skin lesion sizes were measured after 24 h infection, and graphed as mean values ± standard deviation. Data were analyzed with a Student’s t-test and considered statistically different if p≤0.05. (B) Infected skin was harvested 24 h after challenge, and IL-1β, IL-6, KC, and IL-17 levels quantified from homogenates with a 7-plex pro-inflammatory cytokine kit and ELISA kit. Values were normalized to ng/mg total skin lesions, and analyzed with a Dunnett test. Data were considered statistically significant if p≤0.05.

To confirm the role of AT in reducing the proinflammatory mediator response during a *S. aureus* SSTI, mice were infected ID with *Δhla* +/−0.5 µg purified AT or the oligomerization deficient AT mutant AT_H35L_ [14]. Skin lesion size and cytokine levels were then measured 24 h post infection. As expected, infection with *Δhla* alone or in combination with AT_H35L_ did not result in formation of a dermonecrotic lesion however, co-administration of AT with *Δhla* or AT alone resulted in the formation of a >200 mm^2^ lesion (Fig. 4A). Addition of exogenous wild type AT resulted in a significant decrease in IL-1β (p = 0.008), KC and IL-17 (data not shown) in mice infected with *Δhla* when compared to mice that received *Δhla* alone or *Δhla*+AT_H35L_ (Fig. 4B). These results indicate that loss of AT expression is responsible for the diminished immune response and tissue damage seen following infection with *Δhla* and that AT mediated damage is dependent on AT oligomerization and pore formation.

### Anti-AT mAb Reduces Skin Lesion Severity and Facilitates Bacterial Clearance

**Figure 5:**
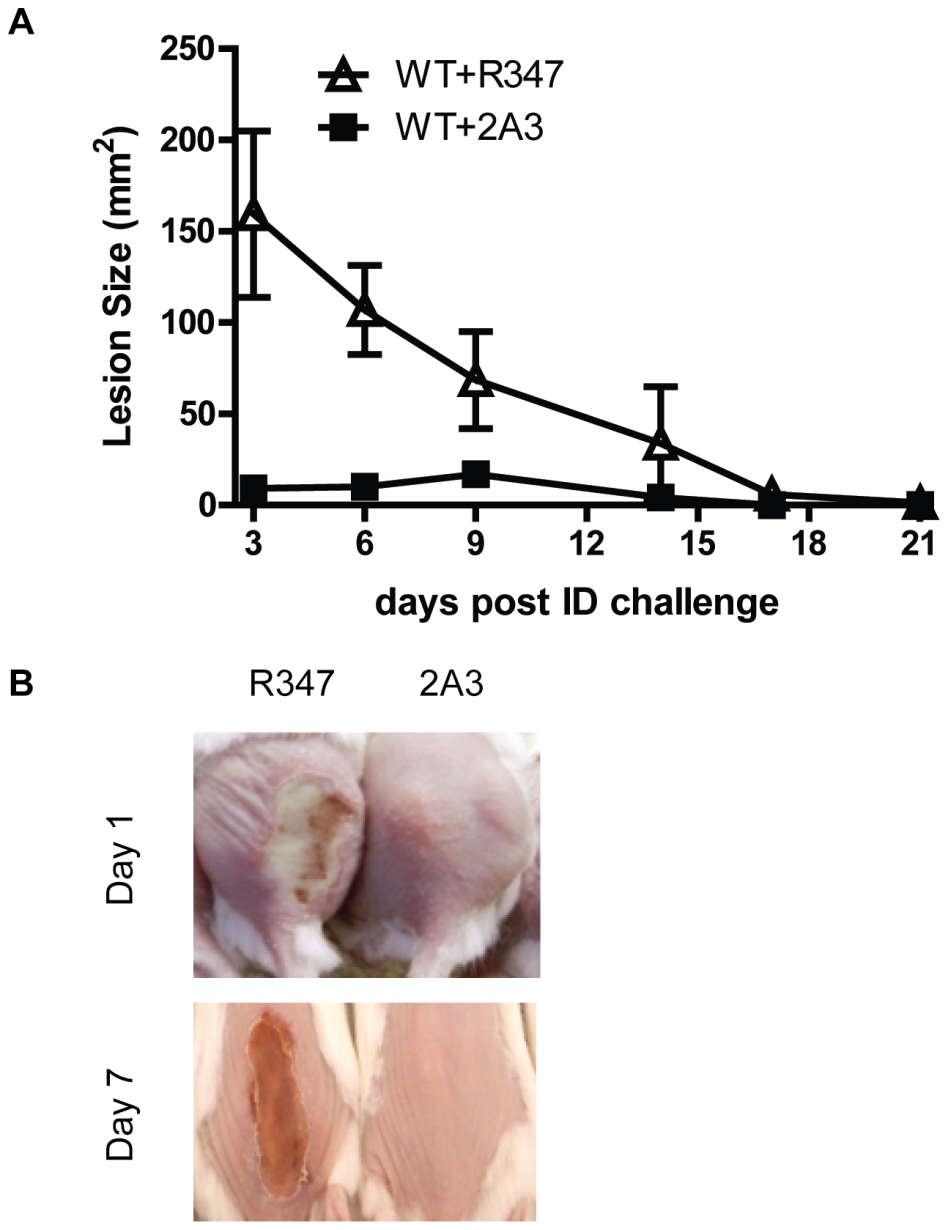
Anti-AT prophylaxis decreases lesion size in *S. aureus* dermonecrosis. BABL/c mice (n = 10) were passively immunized intraperitoneally (IP) anti-AT mAb 2A3 (10 mg/kg) (▪) or the isotype control R347 (Δ), and ID challenged 24 h later with WT SF8300 (5×10^7^ CFU). (A) 2A3 significantly reduced skin lesion sizes. Graph represents lesion sizes up to 21 days post infection as mean values ± standard error. (B) Pictures are representative of mouse skin lesions 1 or 7 day post infection.

**Figure 6:**
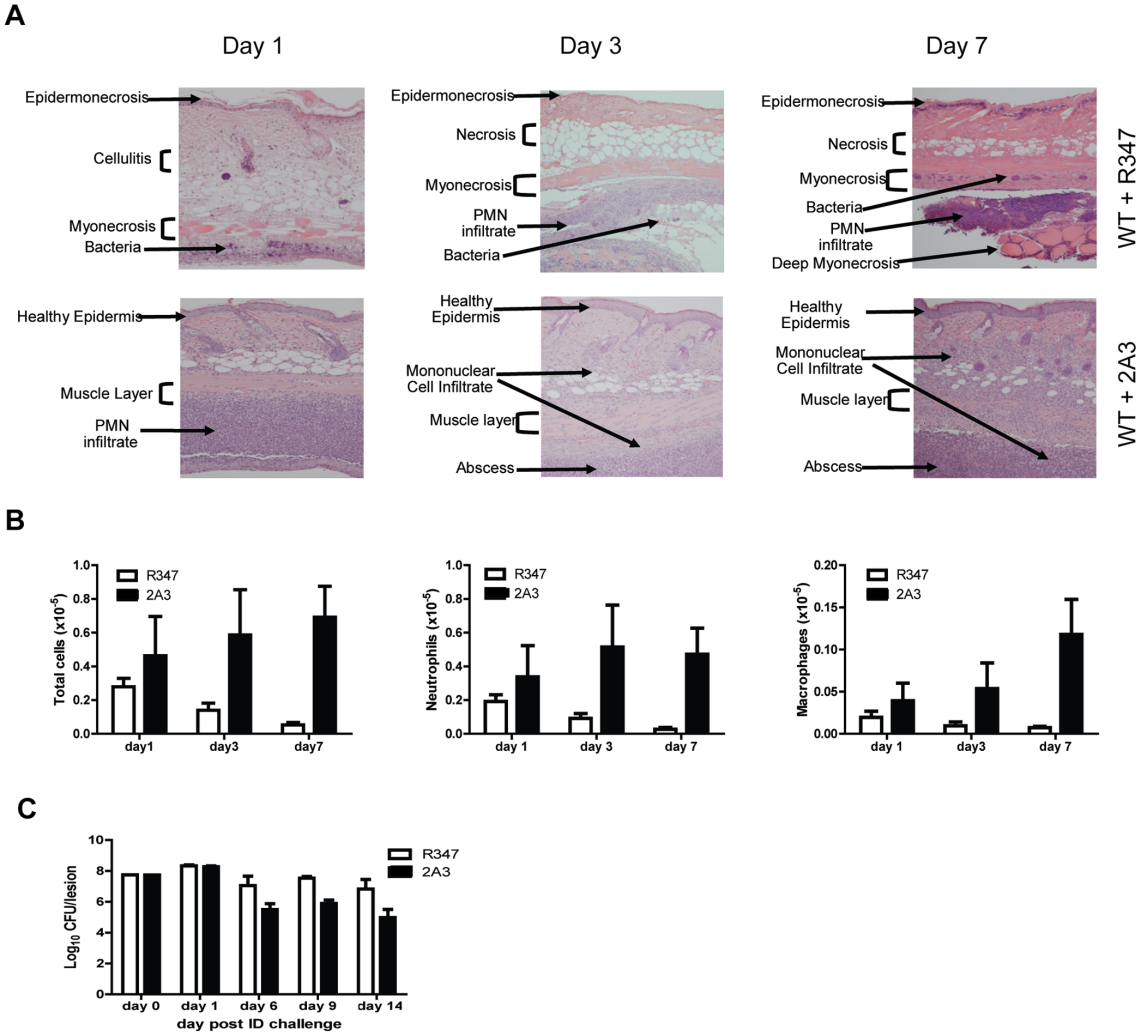
Anti-AT prophylaxis facilitates protective abscess formation and reduces bacterial load. BALB/c mice (n = 10) were passively immunized IP with 10 mg/kg anti-AT mAb 2A3 (▪) or the isotype control R347 (Δ), and ID challenged 24 h later with WT SF8300 (5×10^7^ CFU). (A) 2A3 promotes abscess formation. Representative H&E stained histological section showing mouse infected skin 1, 3 and 7 days post infection. (B) BALB/c mice (n = 5 on days 1 and, n-10 on day 7) were passively immunized IP with R347 or 2A3 (10 mg/kg), followed by ID infection with WT 24 h later. Skin lesions were collected 1, 3 or 7 days post-infection and the neutrophils and macrophages were enumerated by flow cytometry as described in the methods. Data are represented as mean values ± standard deviation. Cell number differences between 2A3 and R347 were analyzed with a Student’s t-test, and considered statistically difference if p<0.05 (indicated as *). (C) Anti-AT mAb facilitates bacterial clearance. Skin lesions were harvested and homogenized for bacteria enumeration 1, 6, 9 and 14 days post infection (n = 5 on days 1 and 6, n = 10 on days 9 and 14). Data were analyzed using a Mann-Whitney U test, and were statically significant after day 6 (p≤0.006).

We previously described a high affinity AT neutralizing mAb, 2A3, that significantly reduced skin lesion size in a mouse dermonecrosis model when administered 24 h prior to ID challenge [14]. Due to the striking differences in the immune response and disease outcome in mice infected with WT +/− AT, we next examined if AT neutralization with 2A3 could influence the host immune response similar to what was observed with *Δhla*. Indeed, 2A3 prophylaxis resulted in >90% reduction in lesion size relative to an IgG1 isotype control, R347 (Fig. 5A, B). Similar to infection with Δ*hla*, 2A3 prophylaxis promoted early neutrophil recruitment and abscess formation resulting in a significant reduction (p≤0.006) in bacterial load 6 days post-infection relative to animals that received R347 (Fig. 2 and 6). These results indicate that prophylaxis with 2A3 significantly reduces disease severity, promotes abscess formation and reduces bacterial load in a *S. aureus* dermonecrosis model.

### AT Neutralization with 2A3 Modulates Pro-inflammatory Mediator Production in Skin Lesions

**Figure 7:**
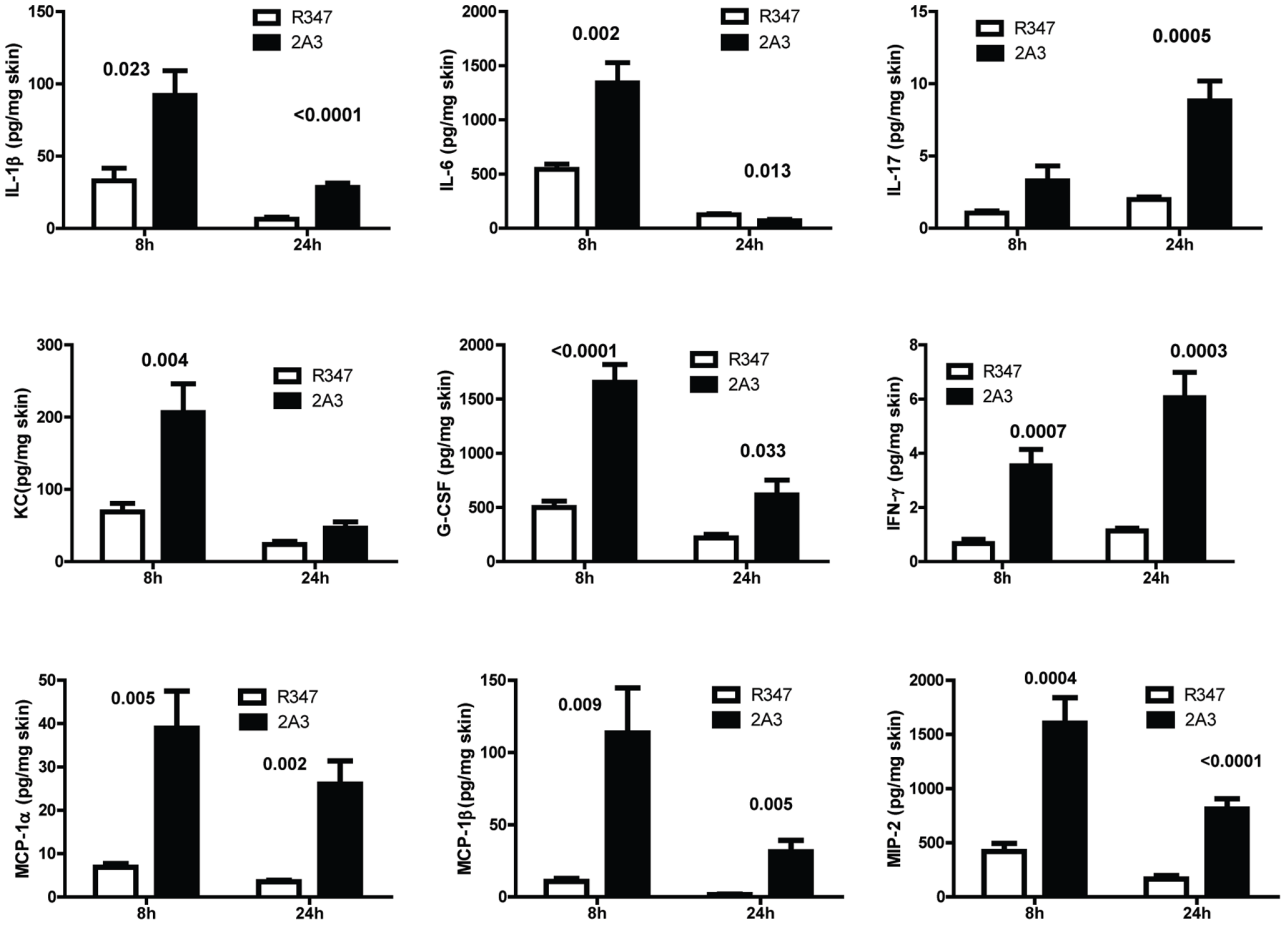
Anti-AT prophylaxis increases pro-inflammatory cytokine and chemokine levels in *S. aureus* infected skin lesions. BALB/c mice (n = 10) were immunized IP with anti-AT mAb 2A3 (▪) or isotype control R347 (□) at 10 mg/kg, and ID challenged 24 h later with WT SF8300 (5×10^7^ CFU). Skin lesions were harvested after 8 or 24 h, and homogenates analyzed by Milliplex for mediator content. Data were normalized to ng/mg total skin lesion. Data were analyzed by Dunnett test, and values were considered statistically different if p≤0.05.

To determine if AT neutralization following 2A3 prophylaxis resulted in a more robust proinflammatory response at the site of infection, cytokine and chemokine levels in skin lesions were analyzed. 2A3 prophylaxis resulted in cytokine and chemokine profiles similar to infection with Δ*hla* (Fig. 7): IL-1β, IL-17, IL-6 and IFN-γ as well as chemokines G-CSF, KC, MIP-2 and MCP-1α/β were significantly increased in skin lesions 8 and/or 24 h post infection relative to lesions from mice that received the R347 control (Fig. 7). Surprisingly, even though the IL-1β levels were similar between Δ*hla* and 2A3+ WT after 24 h, they were significantly higher after 8 h in the 2A3+ WT -injected mice (Fig. 7, p = 0.023). This may result from a small amount of non-Ab neutralized AT.

### AT mAb Increases Localization of IL-17^+^ T Cells and Th1 Cells in Infected Lesions

**Figure 8:**
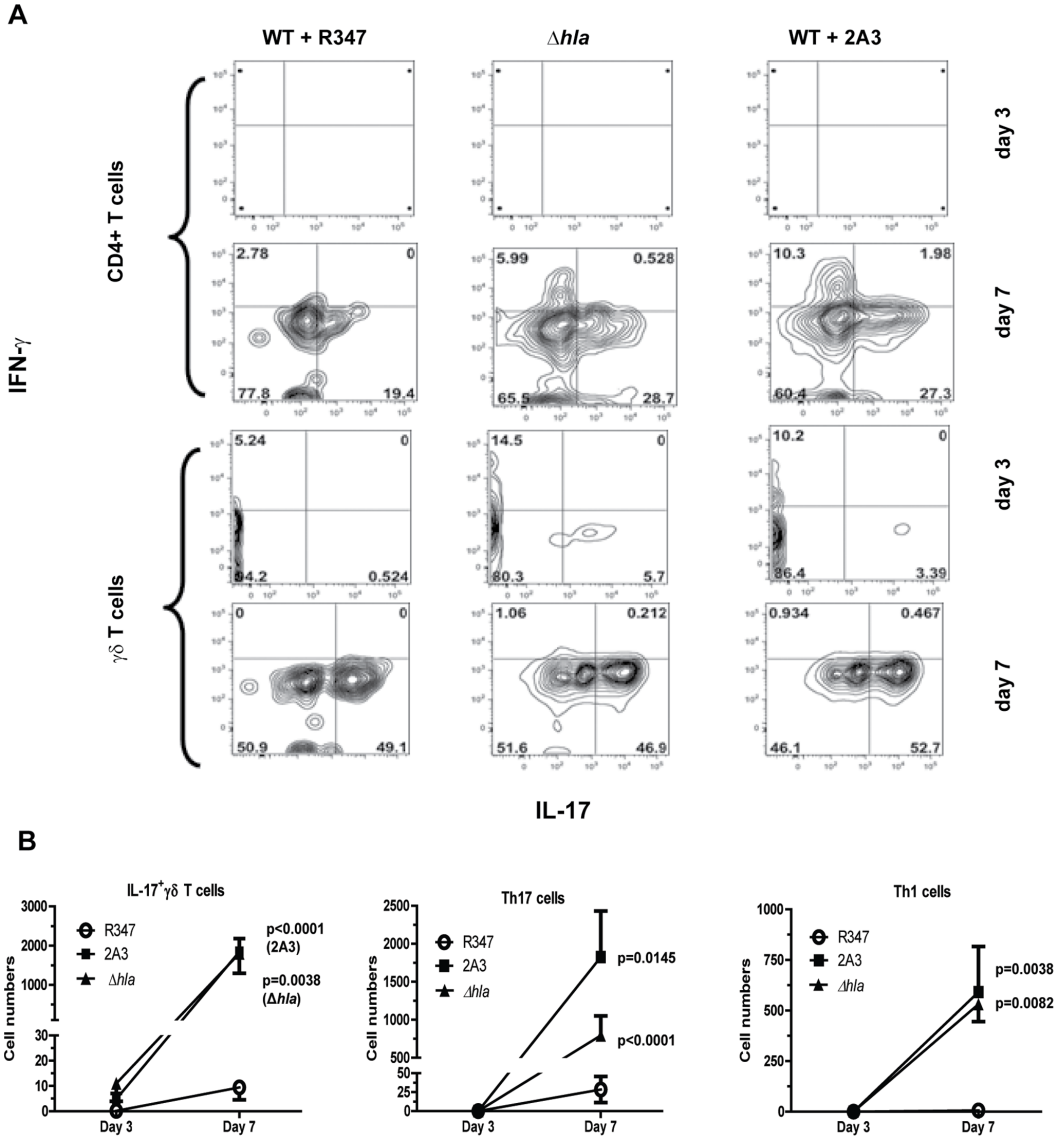
AT delays IL-17 induction in innate γδ T cells and impaired Th17 and Th1 T lymphocyte development. BALB/c mice (n = 5 on days 1 and3, n = 10 on day 7) were passively immunized (IP) with 2A3 or isotype control mAb R347 (10 mg/kg), followed by ID challenge with WT SF8300 (5×10^7^ cfu) 24 h later. As a positive control mice received *Δhla* (5×10^7^ cfu). Skin lesions were harvested 1, 3 and 7 days post challenge, digested with liberase and DNAse. Single cell suspensions were stimulated for 4 h at 37°C with leukocyte activation cocktail containing Golgi Plug (BD Biosciences). Following washing and fixation, the cells were labeled for T cell subsets with anti- CD4, CD8, and anti-β and -γ chain specific Abs. After permeabilization, cells were then incubated with anti-IFN-γ and IL-17 Abs. Cells were gated for TCRβ, or TCRβ^−/^TCRγ^+^ and then for IL-17 and IFN-γ. Intracellular IL-17 and IFN-γ expression was analyzed by flow cytometry. (A) Representative dot plot for IL-17 and IFN-γ intracellular expression in αβCD4+ and γδT cells from one animal in each group on days 3 and 7. The percentage of cells in each quadrant is indicated. (B) Number of IL-17^+^ γδ T cells, Th1 and Th17 cells as determined by flow cytometry 3 or 7 days post-infection. Cell numbers from each mouse were quantified by flow cytometry after specific staining for TCRδ or CD4 in TCRβ^+^ and intracellular staining for IL-17 and IFN-γ. Data are represented as mean values ± standard deviation. Values were analyzed with a Student’s t-test and considered statistically different if p≤0.05.

T cells also play an important role in the host immune response against *S. aureus* cutaneous infections [17], [18], [20], [24] by producing cytokines (e.g. IL-17, IFN-γ and chemokines (e.g. MIP-2 and KC) to promote neutrophil recruitment and activation [20], [25], [26]. To determine the T cell population(s) involved in IL-17 and IFN-γ production during acute infection and later during the healing process, skin lesions were collected 1, 3 and 7 days post WT infection from mice passively immunized with 2A3 or R347. CD4^+^, CD8^+^ and γδT cells were then analyzed for intra-cellular IFN-γ and IL-17 cytokines by flow cytometry. Animals infected with Δ*hla* were included as a control. Although T cell numbers were below our detection limit 1 day post infection (data not shown), IL-17^+^ γδT cells were present in the lesions of mice passively immunized with 2A3 or infected with *Δhla* 3 days post-infection and their numbers significantly increased by day 7. Interestingly, recruitment of this cell population was delayed in mice infected with WT (Fig. 8A, B). Consistent with the initiation of an adaptive immune response, CD4^+^ T cells were not detected until day 7 in any of the mice, however there were significantly more IL-17 and IFN-γ expressing CD4+ T cells on day 7 in the lesions from mice passively immunized with 2A3 or infected with *Δhla* relative to mice that received R347 (see p values on Fig. 8B). Therefore, in this murine dermonecrosis model, AT expression by *S. aureus* delayed recruitment of innate immune IL-17+ γδT cells to the site of infection, and suppressed the adaptive immune response as measured by Th1 and Th17 lymphocyte numbers. Consistent with a role for Il-17 in promoting neutrophil influx and granulopoiesis, increased numbers of IL-17^+^ γδT cells and Th1/Th17 cells in the absence of AT is mirrored by increased neutrophil numbers at the site of infection and enhanced healing (Fig. 2B and 6B).

## Discussion

An effective host response against *S. aureus* skin and soft tissue infection (SSTI) is dependent on IL-1β induced IL-17 expression by resident skin γδ T cells leading to neutrophil recruitment, abscess formation and eventual bacterial clearance [20]. *S. aureus* makes a diverse array of virulence determinants and immune evasion proteins important for disease pathogenesis [27]. Previous studies in mouse and rabbit dermonecrosis models demonstrated AT to be a major virulence determinant in *S. aureus* SSTI. Infection with *S. aureus* mutants defective for AT expression resulted in reduced tissue damage and smaller dermonecrotic lesions relative to WT *S. aureus* [4], [5]. Additionally, mice deficient for expression of the AT receptor, ADAM10, at the site of infection were resistant to infection with WT *S. aureus* and did not exhibit dermonecrosis [13]. However, the effect of AT on the local host immune response was not examined. Using an AT isogenic *S. aureus* USA300 (Δ*hla*) and an AT neutralizing mAb (2A3) we demonstrate for the first time that AT alters the innate and adaptive immune responses during a *S. aureus* SSTI. Mice infected with Δ*hla* mounted a robust immune response resulting in the expression of cytokines and chemokines involved in granulopoiesis, neutrophil recruitment and activation (e.g. IL-1β, IL-17, IL-6, KC, MIP-2, MIP-1α, MIP-β, G-CSF). The robust proinflammatory mediator response in *Δhla* infected mice resulted in immune cell infiltration, abscess formation and increased bacterial clearance. This protective response to wall-off the bacterial infection was suppressed in mice infected with WT or *Δhla*+exogenous AT, resulting in severe tissue damage and a substantially delayed healing process. These results indicate AT directly influences the immune response early during an infection.

Similar to infection with *Δhla*, mice passively immunized with a potent high affinity anti-AT mAb (2A3) prior to WT infection, mounted a robust inflammatory response resulting in abscess formation and reduced disease severity relative to mice that received R347, a nonspecific isotype control. Of note, IL-1β and IL-6 levels were consistently higher 8 h post infection in mice passively immunized with 2A3 compared to animals infected with Δ*hla*. IL-1β production requires two signaling events. One that increases pro-IL-1β levels in the cell and a second signaling event that activates the multiprotein complex called the inflammasome leading to caspase 1 cleavage of pro IL-1β into mature IL-1β [28]. The first signal occurs when pathogen associated molecular patterns (PAMPs) such as lipoteichoic acid, lipoproteins, peptidoglycan or formylated peptides interact with surface expressed pattern recognition receptors (PRR) such as Toll-like receptors (TLR) and the formyl peptide receptor (FPR) or the intracellular PRR NOD2 [29]–[31]. Inflammasome activation leading to caspase 1 cleavage of pro-IL-1β has been shown to be induced by *S. aureus* pore forming toxins such as AT and β and γ hemolysins along with bacterial lipoproteins [32]. In particular, AT pore formation has been reported to induce IL-1β expression through an activation of caspase-1 via ASC/NLRP3 inflammasome [33], and by facilitating entry of *S. aureus* PAMPs into cells for recognition by NOD2, resulting in IL-1β amplified production of IL-6 [34]. Although 2A3 effectively prevents disease and facilitates the induction of a protective immune response, it is plausible that a small amount of residual free AT present in passively immunized mice can lead to NLRP3 and/or NOD2 activation resulting in increased expression of some cytokines (eg. IL-1β and IL-6) in the 2A3 treated animals over what was detected in Δ*hla* infected mice.

Cho et al recently reported *S. aureus* induced IL-1β expression by PMN during dermonecrosis to be dependent on several PRR and AT [23]. However, this was only determined *in vitro* by stimulation of mouse PMNs with AT and *S. aureus* in presence of a neutralizing anti-AT IgG, and not by infecting animals with a strain deficient for AT. This is in contrast with our data in which mice infected with *S. aureus* in the absence of any AT (Δ*hla* infection) mount a robust proinflammatory response. These results suggest that, in addition to AT, other factors elicited by *S. aureus in vivo* either directly or through interactions with local tissue act to induce IL-1β expression. Such factors produced by *S. aureus* that could play a role in inflammasome activation include β and γ hemolysins and lysozyme degraded peptidoglycan [32]. It is also possible that a local stress response to the infection with Δ*hla* causes cells to release ATP resulting in inflammasome activation via the P2X7 purinergic receptor [35], [36]. Although the exact mechanism is not clear it is apparent that factors in addition to AT also play a role in inflammasome activation and IL-1β expression.

In addition to PMN, T cells are important in the immune response against *S. aureus* skin infections. This is supported by the finding that people with low T cell numbers exhibit increased susceptibility to cutaneous *S. aureus* infections [37]–[39]. Moreover, a robust Th1/Th17 response correlates with protection against *S. aureus* infections [24], [40]. During a *S. aureus* SSTI, resident γδT lymphocytes represent an innate source of IL-17 early in infection and Th17 cells a later source during the adaptive immune response [20], [41]. This IL-17 dependent response is particularly important for immunity against SSTI since subjects with atopic dermatitis exhibit increased infection frequency and severity associated with a diminished Th17 response [42], [43]. Additionally, patients with a genetic defect in STAT3 that results in an absence of Th17 cells are more susceptible to *S. aureus* skin infections, again demonstrating an important role for Th17 cells in immune defense against *S. aureus* cutaneous infections [37], [38]. Infection with Δ*hla* or 2A3 prophylaxis prior to WT infection resulted in an induction of IL-17 producing γδT cells 3 days after challenge, and promoted development of Th1 and Th17 cells 7 days post infection relative to animals infected with WT in the absence of 2A3. These results indicate AT expression diminishes both the innate γδT cell response and adaptive Th1 and Th17 responses. IL-17 and IFN-γ have been reported to induce chemokine production resulting in neutrophil and phagocytic functional activation [18], [25], [41]. It is likely that AT neutralization by 2A3 results in increased IL-17 and IL-1β production, leading to increased levels of MIP-2α MCP-1α and G-CSF thereby resulting in neutrophil abscess formation and bacterial clearance. Our results support those published by Cho et al., demonstrating Vγ5+ γδT cells to be a source of IL-17 early during abscess formation in response to *S. aureus* SSTI [20]. In addition, we demonstrate that during a protective response the IL-17^+^ γδT cell levels continue to increase out to day 7 and that an adaptive immune response involving Th1 and Th17 cells begins within 7 days after infection providing further evidence for a role of T-cells in mounting an effective response against *S. aureus* infection.

Neutralization of AT with 2A3 during a *S. aureus* SSTI results in an immune response that is fundamentally different from what has been described when AT is neutralized with IgG during *S. aureus* pneumonia. In a *S. aureus* pneumonia model, infected mice exhibit a robust proinflammatory response leading to PMN infiltration, tissue damage and a consolidating pneumonia [44]. Mice passively immunized with anti-AT IgG prior to intranasal infection with *S. aureus* exhibited reduced IL-1β secretion and increased IFN-γ production in sera, leading to reduced PMN infiltration, improved lung pathology and ultimately survival [7]. The contrasting effect of AT neutralization on the immune response in these two infection models highlights how the immune system must respond differentially depending on the site of infection. In the skin, a robust inflammatory response is necessary and desirable to wall off and control an infection [15], [16]. However, during a lung infection, a similarly robust inflammatory response is deleterious leading to many of the hallmarks of pneumonia [44], [45]. The exact mechanism that leads to the differential immune response to AT depending on the site of infection has not been elucidated.

The mechanism by which AT alters the immune response during dermonecrosis is unclear. It has been reported that AT can kill immune effector cells (e.g. T-cells, monocytes, and peripheral blood lymphocytes) by direct lysis or apoptosis which could play a role in the AT-mediated suppression of the immune response [46]–[48]. Other cells that could be targeted by AT such as mast cells, neutrophils and keratinocytes have also been reported to help orchestrate a protective immune response during bacterial skin infections through the initiation of an innate response via IL-1β secretion [23], [49], [50]. Although direct lysis of cells involved in orchestrating the immune response likely has a role in the immunosuppressive effect of AT, there is evidence this is not the only mechanism by which AT is acting. For instance, on the edges of the lesion where AT is likely lowest there is no immune cell infiltration 24 hr post infection. Moreover, when neutrophils have infiltrated tissue and been killed or lysed there are typically neutrophil remnants present in the form of neutrophilic nuclear dust or neutrophil DNA {{403 Kumar, Vinay 2010}}. There was no evidence of this in wild type SF8300 infected mice. Additionally, AT has been implicated in increasing proinflammatory cytokine expression and immune cell infiltration leading to *S. aureus* pneumonia [51]. In contrast to dermonecrosis, during *S. aureus* pneumonia when AT is neutralized by active or passive immunization or following infection with Δ*hla* the cytokine response is altered and immune cellular infiltration is substantially reduced relative to infection with WT *S. aureus* alone [7], [52]. In the lung, AT acts to increase cytokine expression and promote immune cell infiltration, not lysis. If AT was acting solely through direct lysis of immune cells in the skin it would be expected to have a similar effect in the lung, but it does not. The exact mechanism by which AT diminishes the inflammatory response in the skin is unknown and requires further investigation.

Our results suggest for the first time that, in addition to causing tissue damage during a serious *S. aureus* skin infection, AT also acts to suppress a tissue appropriate protective host immune response, and this effect can be blocked with an AT neutralizing mAb. AT neutralization with 2A3 enhanced the inflammatory immune response resulting not only in IL-17^+^ γδT cell, PMN and macrophage recruitment to the site of infection, but also allowing the immune system to mount an adaptive immune response by inducing Th1 and Th17 lymphocyte differentiation thereby facilitating bacterial clearance and wound healing. Due to its role in multiple aspects of *S. aureus* disease, such as cell lysis, tight junction cleavage and immune dysregulation, AT is a prime target for prophylaxis or treatment against *S. aureus* disease.

## Materials and Methods

### Ethics Statement

Protocols requiring the use of animals in these studies were reviewed and approved by MedImmune’s Institutional Animal Care and Use Committee and comply with the animal welfare standards of the USDA, Guide for the Care and Use of Laboratory Animals, and AAALAC international. (Protocol - MI-12-0016).

### Bacterial Strains and Culture Conditions

CA-MRSA USA300 SF8300 wild type (WT) and its isogenic AT mutant, Δ*hla*, were kindly provided by Dr Binh Diep (University of California, San Francisco). Bacteria were grown to mid-log phase (OD_600_, 0.8) in trypticase soy broth (TSB, VWR International), washed twice in ice cold PBS (Invitrogen), and frozen in 10% glycerol-TSB aliquots. Challenge inocula were prepared from one frozen vial for each experiment, diluted in ice cold PBS at 1×10^9^/ml, and placed on ice until injection.

### Mouse Dermonecrosis Model

The backs of 6–8 week old female BALB/c mice (Harlan) were shaved (n = 10), and treated with Nair (Church & Dwight**)** to remove residual hair. 2A3 and isotype control mAb, R347 were previously described [14]. Antibodies were delivered by intraperitoneal (IP) injection twenty-four hours prior to intradermal (ID) injection with a 50 µl *S. aureus* suspension (1×10^8^ CFU/ml). The animals were monitored twice daily for signs of infection and the abscess size measured once daily. The areas of the lesions were calculated using the formula A = L x W. Statistical significance was determined using analysis of variance and Dunnett’s post-test.

### Cytokine Evaluation in Infected Skin

Skin lesions were harvested at the indicated times post infection, weighed and frozen in liquid nitrogen. Each lesion was homogenized with a mortar and pestle at −80°C then digested for 2 h at 4°C in Reporter Lysis Buffer (Promega) supplemented with complete mini protease inhibitor tablets (Roche Diagnostics). Following centrifugation at 4°C, the cytokine containing-supernatants were stored at −80°C until assessment with a 32 panel mouse cytokine/chemokine Milliplex MAP kit (Milliplex, Millipore) or MSD 7-Plex pro-inflammatory mouse cytokines kit (Mesoscale). For some experiments, IL-17 was quantified with an ELISA kit using the manufacturer’s specifications (R&D Systems). Values were normalized to pg/mg skin, and analyzed for statistical differences with an independent samples t-test, or by ANOVA followed by Dennett’s tests when pre-specified treatments were compared to one control group. SAS 9.1.3 was used for the data analyses. Data were considered statistically significant if the p value ≤ 0.05.

### Bacterial Clearance Kinetics

BALB/c mice (n = 10) were passively immunized IP with 10 mg/kg of anti-AT mAb 2A3 or the irrelevant human isotype control, R347, diluted in 500 µl PBS 24 h prior to ID challenge with SF8300. As a control, animals were challenged ID with *Δhla*. At various time points post-infection, individual skin lesions were harvested and placed on ice in a 14 ml polypropylene round bottom tube (VWR International) containing 1 ml ice-cold PBS. The lesions were then homogenized using a polytron, stainless steel homogenizer (Kinemitic) and plated for bacterial enumeration.

Data were analyzed with a Mann-Whitney U test. Values were considered statistically significant if p≤0.05.

### Histopathological Evaluation of Skin Lesions

Mice were sacrificed and skin lesions were removed, fixed in buffered 10% formalin (VWR International) for 24 h, and paraffin embedded (Leica Microsystems). Four µm sections were stained with hematoxylin and eosin (H&E, Mercedes Medical) following standard histopathological techniques. All stained sections were analyzed using a Nikon80i microscope with 10 and 40× objectives. Histo-pictographs were taken using Nikon Digital Sight mounted camera.

### Skin Processing and Cytometry

Skin lesions were harvested 1, 3 or 7 days post-infection and minced with a razorblade in 10 mm Petri dish (VWR International). Tissues were then transferred to 50 ml conical tube and incubated while shaking for 2 h at 37°C with 10 ml Glutamax RPMI 1640 medium (Invitrogen) containing 0.3 mg/ml Liberase TL (Roche) and 0.01% DNase (Sigma) supplemented with 100 U/ml penicillin, and 100 µg/ml streptomycin (Invitrogen), 0.05 mM β-mercaptoethanol (Gibco), and 100 µM non essential amino acid (Gibco). To obtain a single cell suspension, digested tissues were filtered through a 70 µm cell strainer (BD). To lyse red blood cells, the cell pellet was incubated on ice with 3 ml ACK buffer (Invitrogen) for 3 min then washed twice with 50 ml supplemented Glutamax RMPI containing 10% fetal bovine serum (FBS) (Invitrogen). The cells were resuspended in 500 µl in RPMI 10% FBS. One hundred µl were stained for neutrophil/macrophage enumeration; 400 µl were then stimulated for 4 h at 37°C in 5% CO_2_ incubator with 100 ng/mL phorbol myristate acetate (PMA), 2 µM ionomycin (BD Pharmingen) and 1 µg/ml brefeldin A (BD Pharmingen) diluted in 500 µl supplemented Glutamax RPMI media containing 10% FBS. After one wash in supplemented RPMI containing 10% FBS, cells were transferred to a U bottom 96-well plate (VWR), and stained with Hoescht solution to separate dead/live cells. Fc receptors were blocked by incubating the cells with anti-mouse CD16/32 (BD Biosciences, Clone: 2.4G2), followed by fluorochrome-conjugated antibodies diluted in FACS buffer (PBS containing 0.1% FBS). The following fluorochrome conjugated-antibodies were used against the following surface molecules: PerCP-Cy5.5TCR β (eBioscience, Clone: H57-597 ), FITC-TCRγδ (BD Pharmingen, Clone: GL3 ), Pacific blue-CD4 (BD Pharmingen, Clone: RM4-5), APC-H7-CD8 (BD Pharmingen, Clone: 53-6.7). For intra-cellular staining, cells were first fixed with 4% formaldehyde, and permeabilized with 0.1% saponin (BD Pharmingen), followed by incubation with PE anti-IL17 (BD Pharmingen, Clone: TC11-18H10) and APC - anti-IFN-γ (eBioscience, Clone: XMG1.2). Samples were then acquired using a LSR-II flow cytometer (BD), and data analyzed with FlowJo software (Tree Star, Inc.).

### Neutrophil and Macrophage Enumeration

One hundred microliters of skin single cell suspension were first stained with Hoescht for live/dead gating. Samples were then incubated with PE-Cy7-Gr-1b (eBioscience, Clone: RB6-8C5), APC-F4/80 (eBioscience, Clone: BM8), and FITC-CD11b (eBioscience, Clone: M1/70) Abs for 1 h at 4°C. Following wash, samples were acquired using a LSR-II flow cytometer (BD), and data analyzed with FlowJo software (Tree Star, Inc). Percentage of neutrophils and macrophages were calculated respectively as % Gr-1b^+^/CD11b^+^ and %F4/80^+^/CD11b^+^. Total cell numbers for each population was then calculated based on cell counts from initial skin single cell suspension.
